# Empowering the Internet of Vehicles with Multi-RAT 5G Network Slicing

**DOI:** 10.3390/s19143107

**Published:** 2019-07-13

**Authors:** Ramon Sanchez-Iborra, José Santa, Jorge Gallego-Madrid, Stefan Covaci, Antonio Skarmeta

**Affiliations:** 1 Department of Information and Communications Engineering, University of Murcia, 30100 Murcia, Spain; 2 Department of Electronics, Computer Technology and Projects, Technical University of Cartagena, 30202 Cartagena, Spain; 3 Next Generation Networks (AV), Department of Telecommunication Systems, Technische Universität Berlin, 10623 Berlin, Germany

**Keywords:** network slicing, vehicular networks, 5G, ITS, IoV

## Abstract

Internet of Vehicles (IoV) is a hot research niche exploiting the synergy between Cooperative Intelligent Transportation Systems (C-ITS) and the Internet of Things (IoT), which can greatly benefit of the upcoming development of 5G technologies. The variety of end-devices, applications, and Radio Access Technologies (RATs) in IoV calls for new networking schemes that assure the Quality of Service (QoS) demanded by the users. To this end, network slicing techniques enable traffic differentiation with the aim of ensuring flow isolation, resource assignment, and network scalability. This work fills the gap of 5G network slicing for IoV and validates it in a realistic vehicular scenario. It offers an accurate bandwidth control with a full flow-isolation, which is essential for vehicular critical systems. The development is based on a distributed Multi-Access Edge Computing (MEC) architecture, which provides flexibility for the dynamic placement of the Virtualized Network Functions (VNFs) in charge of managing network traffic. The solution is able to integrate heterogeneous radio technologies such as cellular networks and specific IoT communications with potential in the vehicular sector, creating isolated network slices without risking the Core Network (CN) scalability. The validation results demonstrate the framework capabilities of short and predictable slice-creation time, performance/QoS assurance and service scalability of up to one million connected devices.

## 1. Introduction

**Figure 1:**
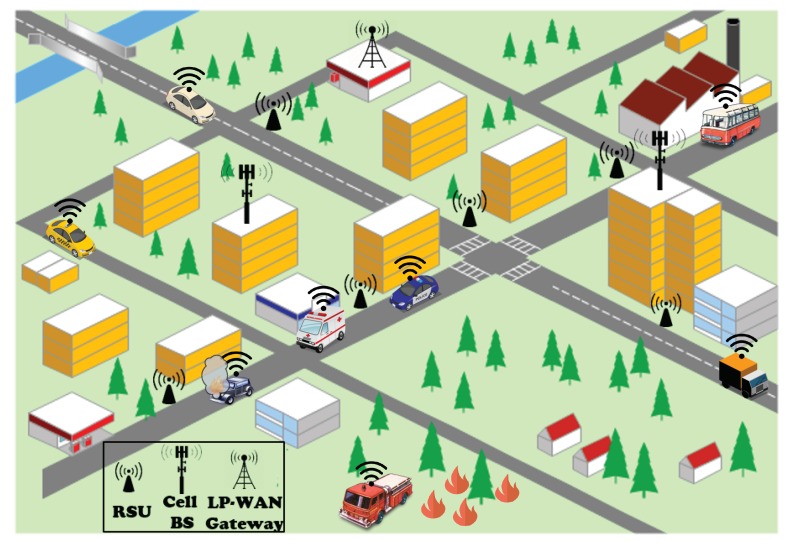
Heterogeneous vehicular scenario (RSU, Road Side Unit; BS, Base Station).

Internet of Vehicles (IoV) will be one of the most benefited vertical industries within the Internet of Things (IoT) ecosystem with the imminent development of 5G architectures [1]. Three main classes of use-cases/services have been already defined for 5G, namely, evolved Mobile Broadband (eMBB), Ultra-Reliable, Low-Latency Communication (URLLC), and massive Machine-Type Communication (mMTC) [2]. In IoV scenarios, the three families of services will coexist, e.g, critical services for road safety or alert notification in URLLC, vehicle and load monitoring in mMTC, and on-board infotainment in eMBB, among others [3]. These services exhibit diverse Quality of Service (QoS) in terms of delivered latency or bandwidth, which is translated to different network demands [4]. Depending on the service under consideration, one Radio Access Technology (RAT) may be more suitable than another for transmitting the associated traffic to/from the Core Network (CN). Examples of RATs that will be available under the 5G umbrella in vehicular scenarios are: Dedicated Short-Range Communication (DSRC) technologies like IEEE OCB (formerly known as 802.11p), cellular networks, and Low Power—Wide Area Networks (LP-WAN), such as LoRaWAN or NB-IoT. For those non-3GPP RATs, the Non-3GPP InterWorking Function (N3WIF) has been defined, which provides them with support for non-3GPP access to the 5G CN [5]. Therefore, there is a clear intention for integrating different-nature RATs within the 5G infrastructure. These communication alternatives present notable differences regarding their coverage or bandwidth characteristics. Figure 1 shows a typical urban scenario with different kinds of vehicles and RATs.

**Figure 2:**
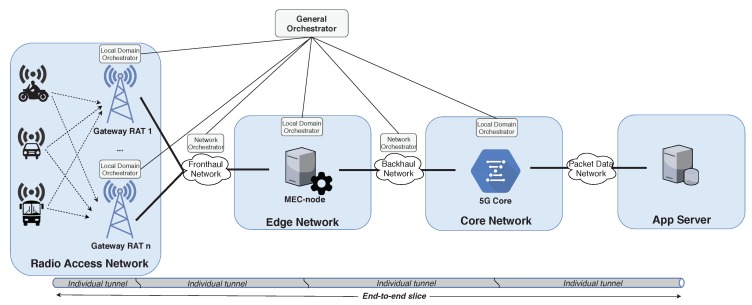
Overall architecture of the multi-RAT 5G slicing framework.

The 5G transport network should be able to differentiate and isolate the different traffic flows crossing the system, as well as guaranteeing a set of network resources to them [6]. This technique, which is known as network slicing, consists in configuring dedicated networks with assured virtualized resources for specific users and/or applications over a common physical infrastructure. Network slicing has emerged as one of the key technologies to support the vast number of heterogeneous vehicular services as those aforementioned, by using a common network management scheme. For that reason, in this work, we present a real implementation of a network slicing framework that empowers the co-existence of heterogeneous IoV applications. As shown in Figure 2, we have adopted a Multi-Access Edge Computing (MEC) architecture in order to begin the slicing process as close as possible to the end-nodes instead of applying it just in the CN and WAN, and to permit the agile distribution of the 5G slicing modules. We integrated our solution with a real 5G core implementation, namely, OpenAirInterface [7], and validated it in a real scenario.

The remainder of the paper is organized as follows. Section 2 reviews related works in the literature and identifies the advances beyond the state of the art. Section 3 presents the general architecture of the network slicing framework. Section 4 details the implementation of the system. Section 5 addresses the validation and evaluation of the platform. Section 6 concludes the paper and proposes future research directions.

## 2. Related Work

Although there are other approaches in the literature dealing with QoS assurance in vehicular networks, such as information-centric proposals powered with features to limit packet delivery time [8], the spread of 5G solutions carries inherent advantages for data flow management. In this section, we explore the most relevant network slicing proposals in frames of the 5G ecosystem to enable the development of novel IoV applications. It is shown the high interest in the research area, although important gaps are found in real implementations, emphasizing IoV adaptation and end-to-end slice management distribution.

Campolo et al. introduced the benefits of network slicing when applied in vehicular scenarios [9,10]. Both works expose the technological solutions and network-enablers for the successful implementation of slicing frameworks aligned with ongoing 3GPP standardization activities, by making use of the most novel network softwarization advances. In this line, the authors highlighted the interaction of Network Function Virtualization (NFV), Software Defined Networks (SDN) and MEC as the keystones for the development of network slicing solutions for Intelligent Transportation Systems (ITS). Besides, the authors identified the main requirements for network slicing systems devoted to vehicular services: support for multi-tenancy services with a massive number of users, transparent mobility for the heterogeneous variety of connected vehicles, slice isolation and security, and dynamic slices providing seamless connectivity to vehicles in different scenarios with diverse connectivity conditions. Within the same research line, the authors dealt with an initial implementation of such slicing framework in [11], especially focusing on the mobility of vehicles under inter-domain handovers in 3GPP networks. Hence, a roaming-capable slicing approach was developed by using the support of SDN functions to assist handovers. A validation of the framework was presented using a network emulator.

Several slicing schemes have been proposed in the related literature but always from analytic or numeric simulation perspectives. Therefore, gaps have been identified related to the real implementation of 5G slicing platforms and their validation in vehicular scenarios. The authors of [12] employed the Euclidean norm theory for proposing a reliability and latency joint function to evaluate the impact of reliability and latency in 5G vehicular networks. From this evaluation, a network slicing solution was proposed in order to improve the system performance in terms of these two parameters. In [13], a cell planning scheme was proposed with the aim of maximizing the RAN resource efficiency while meeting certain Quality of Service (QoS) requirements. The authors employed a convex inner approximation and the simulation results validated this proposal. Another network slicing mechanism was proposed in [14], in this case focusing on mobility management. The authors validated their proposal by demonstrating a reduction in the average cost of processing a mobility event. In [15], a slicing scheme and an optical-network-units migration algorithm for dynamic vehicle communication applications were presented. An improvement in terms of switching times and transmission distance were attained, which lead to maximized bandwidth utilization. The issue of efficiently creating network slices from a given pool of network resources was investigated in [16]. This was studied from an analytic perspective considering it as a combinatorial optimization problem. With this approach, the authors achieved to increase the resource utilization efficiency.

The authors of [17] took advantage of the flexibility gained by virtualizing the Radio Access Network (RAN) in order to develop a slicing mechanism for dynamic vehicular scenarios. In this work, a scheduling algorithm based on a Markov decision process was developed for coordinating the allocation of RAN resources considering the road traffic status. To this end, a collaborative scheduling scheme was presented aiming at adjusting the traffic speed in the distribution of IoV resources. Simulation results showed throughput improvements and reduction in network delay with a limited computational load. Another approach for slicing the RAN segment of the network was presented in [18]. In this case, the authors defined two slices, one for infotainment transmissions and the other for safety messages, and assigned different physical resources to each one. While the former was only supported by the fixed infrastructure, for the second one, some vehicles were selected as dedicated access points for providing high-priority connectivity to others. The connection of vehicles to the fixed infrastructure or other supporting vehicles was managed by considering physical connectivity parameters such as the Signal to Interference and Noise Ratio (SINR). With this scheme, the authors showed improved levels of throughput in both slices extracted from simulation experiments. Finally, a network slicing framework for both wired and wireless domains in 5G was presented in [19]. Similar to the work in [16], a network utility maximization problem was formulated to determine the optimal bandwidth assigned to the different slices. This strategy permitted reducing the delay in packet queues, hence improving the performance of the transported services.

As stated above, the validations of the proposals reviewed above lack in providing end-to-end slicing scenarios with a proper implementation using new-age RATs. Moreover, concrete proposals are not always extensible to any new RAT, lacking generic slicing frameworks. The solutions reviewed also present the drawback of including evaluations through simulation or analytical studies. Differently from these works, we present in this paper the real implementation and validation of a multi-RAT framework for 5G slicing in vehicular scenarios. In addition, none of the proposed solutions so far has adopted a MEC architecture to achieve a close-to-RAT approximation with extra management and performance capabilities, and the packet marking procedure based on differentiated services is a non-common approximation that provides good performances. With no doubt, these features provide the system with a notable flexibility in order to distribute slicing capabilities along the network path, supported with the addition of platform functionalities as VNFs.

## 3. General Architecture

The general architecture of the proposed slicing framework is presented in Figure 2. All the slice lifecycle operations are managed by the General Orchestrator (GO), which is in charge of configuring and (re)deploying the slices by communicating with the local Domain Orchestrators (DO) placed in all the entities of the architecture, namely, RAN-node, MEC-node, and CN. Focusing on the uplink direction, when a slice creation request is received from an on-board communication unit, it is forwarded to the GO, which checks if the request can be granted and under what conditions. If the request is accepted, the GO commands to the elements in the path between the end-device and the application server to deploy individual tunnels with the required QoS parameters to transport the traffic flows associated to this slice. Once the slice is configured, the vehicle is informed about the QoS Flow ID (QFI) assigned to the dedicated slice instance (tunnel) and the data transmission may start. More details about this process are given in Section 4.

The proposed slicing scheme ensures flow isolation by individually tunneling data traffic from the MEC-node to the app-server. The particular QoS treatment for each flow begins at the MEC-node where the flows are prioritized according to their nature and the QoS rules associated to each slice are applied. The MEC-node also marks the packets (Differentiated Services Code Point (DSCP) marking) before their forwarding to the 5G’s CN. This is done to ensure the scalability of the CN, since many MEC-nodes may be distributed horizontally over the architecture, but a unique CN is in charge of giving service to a great number of devices or applications. Therefore, it is not feasible to deploy individual bearers for each flow within the CN as it was done in 4G networks. For that reason, we have adopted an approach similar to the Differentiated Services (DiffServ) Per Hop Behavior (PHB) in the CN to prioritize traffic flows based on packet’s marking. With this strategy, we ensure the system scalability upon the connection of new users or devices, which will be a reality in the IoV domain with connected vehicle sensor units, on-board personal devices and interaction with pedestrians. Therefore, the main design objectives of the proposed 5G slicing framework are: (i) bandwidth control and assurance; (ii) flow isolation; (iii) MEC-based traffic shaping and prioritization following the 5G QoS Identifiers (5QI) defined in [20]; and (iv) real on-demand slicing over a 5G CN.

Finally, it is important to remark that the different blocks forming the slicing solution are treated as individual micro-services encapsulated as VNFs, thus assuring the modularity of our solution. They may be distributed in the different entities along the end-to-end path according to the network topology and available resources.

## 4. Structure and Working Flow of the Solution

This section provides detailed descriptions of the modules of the general architecture, together with their interactions.

### 4.1. Orchestration

**Figure 3:**
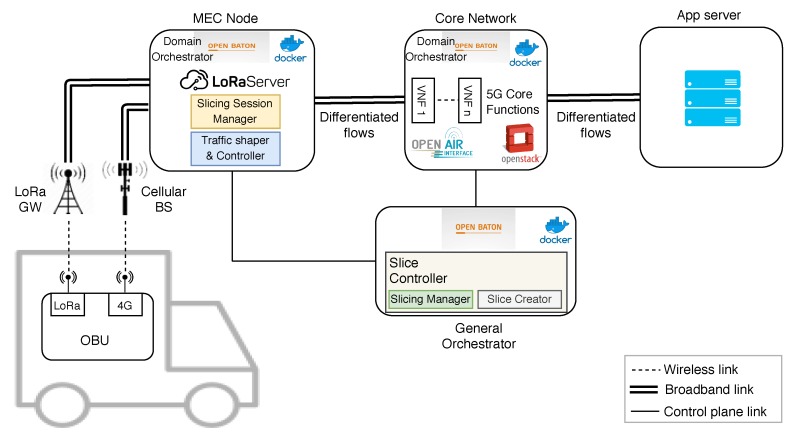
Implemented slicing framework for vehicular applications.

Our proposal presents two orchestration tiers, compliant with ETSI NFV-Management and Orchestration (MANO): the GO and the DOs (Figure 3). The former is placed on the top of the system hierarchy in order to have control over the deployed slices. It consists of an OpenBaton [21] instance and of two micro-services in the form of VNFs: the Slicing Manager (SM) and the Slice Creator (SCr). The SM processes the slice-creation requests sent by the Slice Session Manager (SSM) placed in the MEC-node. When a request is received, the SM checks the requester subscription stored in the 5G CN’s Unified Data Repository (UDR); this functionality is equivalent to the one described for the Network Slice Selection Function (NSSF) in [20]. To this end, we have developed a specific implementation and interface of the UDR, hence enabling this information exchange. If the slice request is accepted, the SM assigns a new QFI to the new slice to be instantiated and sends to the SCr a tunnel-creation request with the associated QoS parameters. To do so, the SM has access to a slice-catalog in which a series of slice templates are defined. From this catalog, it finds the option that better fits the requester’s QoS parameters, which are defined in its subscription stored in the UDR. Once the SCr receives the slice-creation request from the SM, it communicates with each of the DOs to indicate the forwarding and QoS rules to be enforced. The DOs communicate with the different elements of the architecture involved in the deployment of the individual tunnels through two developed plugins: (i) one for the MEC-node, which deploys a tunnel with specific QoS characteristics; and (ii) another for the CN, which establishes a tunnel with QoS in the CN’s UPF towards the app service.

### 4.2. Radio Access Network

In our deployment, we have considered two different RATs: 4G, which is a mobile broadband technology, and LoRaWAN, which is a long-range low-bandwidth technology. The end-device selects the RAT that is more adequate depending on the characteristics of the application-traffic to be sent. We have defined several traffic classes and, attending to their demands in terms of latency and bandwidth, we send them through one radio interface or the other. When receiving data flows, the gateways/base stations forward this traffic to the corresponding entry point in the MEC node, where the QoS differentiation begins.

### 4.3. MEC-Node

This module hosts a series of VNFs with different functionalities. The SSM is in charge of managing the slices’ sessions and, in the case of being necessary, requesting the SC the deployment of a new one. It controls the life-time validity of each instantiated slice. The SSM is also the contact point of a given slice requester with the slicing framework. In turn, the Traffic shaper and Controller (TC) is a VNF devoted to shape and control each Service Data Flow (SDF). Two different implementations of this module have been developed. The first one is based on the native Linux network stack, so the slicing rules are directly applied to the native Linux packet scheduler and filter. The other alternative is employing an Open vSwitch (OvS) as traffic shaper and controller, by using OpenFlow from the SCr to inject the corresponding forwarding and QoS rules. Note that, no matter the selected option, both of them allow defining fixed thresholds for certain QoS metrics such as minimum or ceil bandwidth (bps), among others. After several tests, we have adopted the first approach, given its greater efficiency and versatility for handling flows with different strict QoS requirements. Additionally, the MEC-node hosts an instance of the LoRaServer, which enables communication with the LoRaWAN RAN. It acts as a bridge between the IP and LoRaWAN segments of the network.

### 4.4. Core Network

The OpenAirInterface Release 3 has been deployed in the core network, tuning it in order to enable its interoperability with the proposed slicing framework. Concretely, the interactions are performed in two different ways. First, the SM needs to extract information from the 5G’s UDR regarding the slicing and QoS subscription of the slice requester when a slice is requested. Thereafter, the SCr should configure a path through the UPF in order to establish the slice instance towards the app-server. For this to be done, we have developed two interfaces that permit the related message exchanges. The first one permits to send requests to the database within the UDR in order to retrieve the slice-requester’s subscription data. The other interface permits the SCr to inject the flow-configuration to the UPF elements by means of OpenFlow. As in the TS function in the MEC-node, the latter permits to define minimum and maximum data-rates for each QoS class, as well as tuning other QoS parameters, in the UPF. The rest of the OpenAirInterface modules have remained untouched and we assume that the slice-requesters have been previously registered in the 5G system.

### 4.5. App-Server

This block does not present any specific characteristic related to network slicing. It hosts the services in charge of receiving and processing the data generated by the end-devices in the IoV scenario. In our deployment, we have assumed that the app-server is installed within the network-service provider domain/premises. With this configuration, we have a thorough control over the different domains that the slice instance has to traverse. In the case of having cloud servers hosted outside the Telco’s network, e.g., on the Internet, the DSCP packet marking performed in the MEC-node may be employed for prioritizing the diverse traffic classes in the different management domains crossed until their final destination. This approach has been adopted by Cisco in its recently proposed Software Defined–Wide Area Network (SD-WAN) [22].

### 4.6. Working Flow

**Figure 4:**
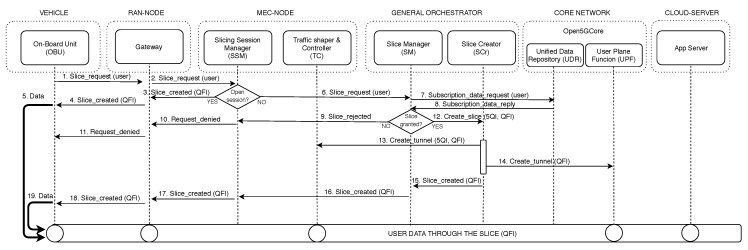
Interaction work flow among components.

Figure 4 represents the interactions among the different blocks that compose the slicing framework. The slicing configuration process begins with a request from an end-device (1), which is forwarded by the corresponding gateway to the SSM (2). This module checks if there is any active session for the requester and, in this case, the QFI of this session is sent back to the requester (3 and 4); hence, the on-board device can continue sending data traffic through this tunnel (5). In the case of not having an active session, the slice request is forwarded to the SM over an open pre-deployed tunnel devoted to transport control traffic (6), which retrieves the requester’s subscription data from the UDR (7 and 8) and checks it. If the request is rejected, the requester is informed about this issue (9–11). If the request is accepted, the SM assigns a new QFI to the new slice to be created and commands the SCr to configure a new slice instance (12). For this to be done, the SCr sends the forwarding and QoS rules to the different involved blocks through their respective DOs (13 and 14). Once these entities acknowledge the SCr the correct enforcement of the tunneling rules (not shown for simplification), it confirms the SM the readiness of the tunnel (15). Finally, the SM provides the requester through the SSM (16) with the QFI assigned to the configured slice (17 and 18), so the requester may begin to send traffic though it (19). Note that in Figure 4 the circles in the configured slice represents contact points of the data-plane tunnel with the different blocks of the architecture.

## 5. Validation and Evaluation

The slicing platform presented above was deployed and evaluated in a real deployment. The performance of the solution was tested by analyzing its operation with traffic flows of different classes.

### 5.1. Implementation and Test Description

As foundation technologies for the instantiation of the system components in virtual infrastructures, Docker and OpenStack have been adopted as container engine and Virtualized Infrastructure Manager (VIM), respectively. The main blocks of the architecture, i.e., MEC-node, CN, and app-server, have been deployed on individual compute-node virtual instances (Ubuntu Server 16.04) over an OpenStack environment. VNFs have been coded in Python and encapsulated as Docker containers. All these elements are managed by the OpenBaton orchestrator, so they can be launched or scaled on-demand along the different parts of the infrastructure (functionality not addressed in this work).

For the validation test, a car was equipped with an On-Board Unit (OBU). It is a Commsignia’s Laguna LGN-20 unit provided with a TP-LINK MA-180 modem for 4G connectivity. Besides, by using the Unix interface for LP-WAN technologies presented in [23], the OBU was also connected to an Arduino board that integrates an RN2483 radio module from Microchip. It operates in LoRaWAN Class A mode, operating in the ISM 868 MHz band. The LoRaWAN gateway used is a RisingHF RHF2S008, which incorporates the Semtech’s SX1301 chip. With this configuration, the OBU was enabled to send traffic seamlessly to both interfaces. A series of traffic classes was defined and, attending to their network requirements, the corresponding traffic flows were re-directed internally in the OBU’s operative system to be sent by one interface or the other.

For the performance evaluation, the RAN block of the network was emulated to focus on the slicing framework itself and for having more flexibility when configuring the testing scenarios. For the LoRa segment, we employed the Lorhammer (http://lorhammer.itk.fr/) tool, which can act on behalf a certain number of end-devices connected to the LoRaServer and generate traffic at a configurable rate. For 4G, several traffic flows with different bit-rates were injected in the system. As mentioned above, the 5G Core was implemented by using the latest version of the OpenAirInterface toolkit.

Several stress-tests were conducted to study the system response under diverse load conditions. To this end, different SDFs with different priorities were injected and some Key Performance Indicators (KPI) were monitored and evaluated. Concretely, we measured the latency when configuring a slice and the throughput and end-to-end latency of the data flows when assigned to different-priority slices.

### 5.2. Validation of the Solution

**Figure 5:**
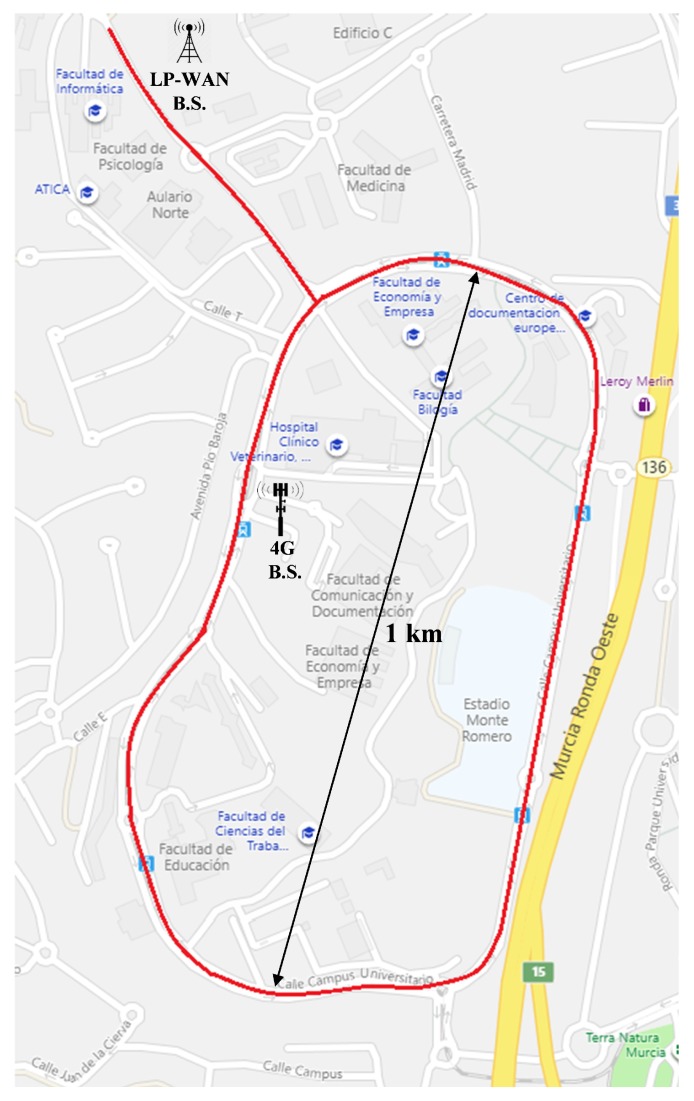
Experimental scenario for the validation test.

The platform was validated by means of an experimental test in which different data flows were sent from the car to an app-server placed in a private cloud, as shown in Figure 3. The chosen scenario for this trial was located at the Espinardo Campus of the University of Murcia (Spain), which was covered by the LoRaWAN gateway and a 4G Base Station (BS) employed for providing ubiquitous connectivity along the campus (Figure 5). As explained above, the LoRaWAN gateway was deployed and managed by us; however, as we employed a 4G cellular network handled by an external Telco, we configured a Virtual Private Network (VPN) tunnel from the OBU to the MEC-node.

In this experiment, the OBU transmitted three simultaneous traffic flows. The first one was devoted to transport discontinuous monitoring data though the LoRaWAN interface at a rate of one message per second. In this case, we transmitted the car speed, measured by the GPS module of the OBU, but could include any other telemetry data common in monitoring services, or sensor reports within constrained scenarios with battery powered vehicles such as e-scooters or e-bikes. This data flow was assigned to a network slice without bandwidth restrictions. The others flows consisted of constant UDP traffic generated by two independent *iPerf* processes that were sent to the app-server through the 4G network, aiming at validating the bandwidth-limitation capability of our solution under more demanding services. The first UDP flow was assigned to a slice with a maximum bandwidth of 1 Mbps; in turn, the second constant flow was sent through a slice with a maximum data-rate of 512 kbps. Both figures were notably lower than the maximum bandwidth provided by the 4G RAT for each flow.

**Figure 6:**
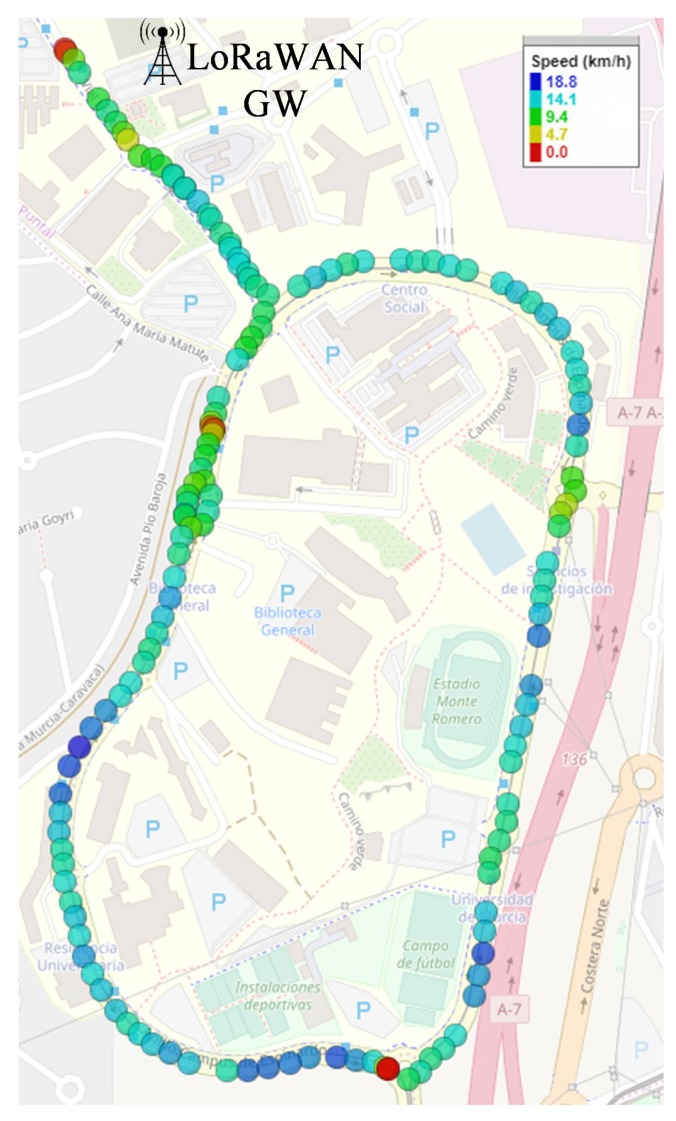
LoRaWAN slice validation test.

**Figure 7:**
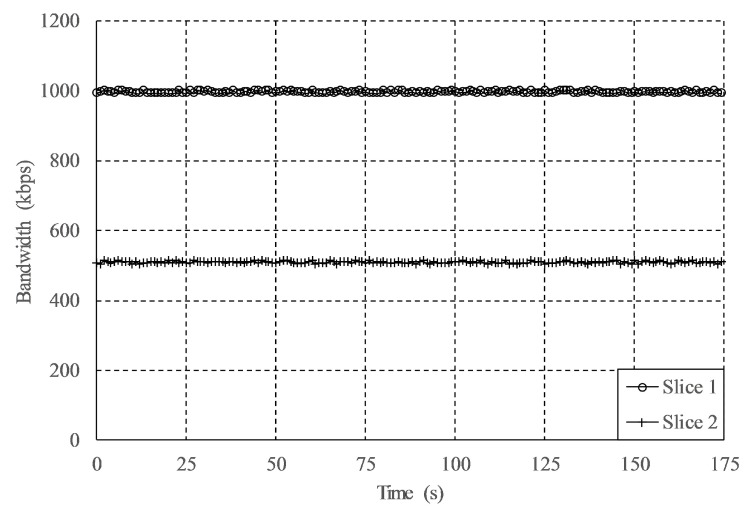
Bandwidth control for isolated slices in the validation test.

Figure 6 presents the instantaneous vehicle speed measured by the OBU’s GPS module and transmitted by means of the LoRa technology. The figure shows that the whole campus is covered by this RAT and that no shadow areas were detected. Some sporadic packet losses were observed, represented as blank spaces between colored dots. These losses are not related to the slicing system but to the RAT, at locations where direct line of sight is not possible due to nearby buildings. For the constant data-rate transmissions through 4G, Figure 7 depicts the data-rate evolution on each of the two defined slices. Observe that data flows with bandwidths of 1 Mbps and 512 kbps are impacted by mobility, as can be seen in the minimal bandwidth fluctuations, but a constant bit rate is achieved. In light of these results, the operation of the slicing framework is considered to be validated, hence the following part is focused on more challenging tests that evaluate the capabilities of the proposed solution.

### 5.3. Performance Analysis

**Figure 8:**
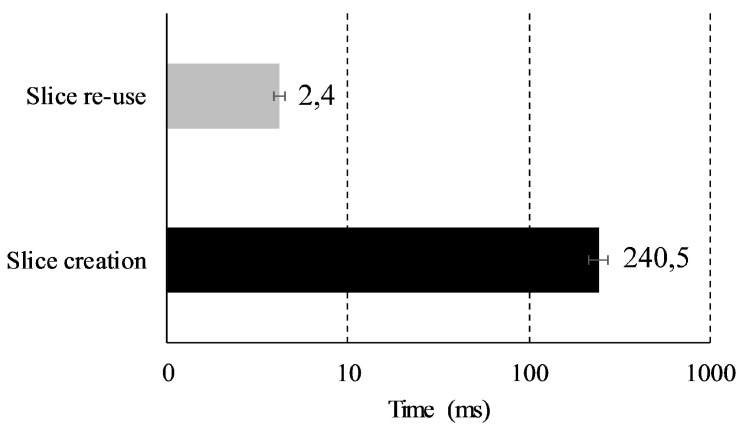
Delay when configuring a slice.

A deeper study was conducted for evaluating the system’s response under different configurations. Figure 8 depicts the average delay, measured since a slice request is received by the SSM until it replies the requester with the corresponding QFI of the assigned slice. In the case the requester already had an active session, the system only sends back the QFI assigned to the slice instance, hence the requester may immediately begin the data transmission. This is a quick procedure that just takes 2.4 ms. In turn, if a new slice has to be instantiated, the whole slice configuration process explained above is triggered. Although the needed time is longer that in the previous case, this is less than 250 ms. Therefore, the framework is capable of configuring a slice for a given requester in a very short time; the attained figures seem to be highly competitive in comparison with other slicing frameworks [24]. Besides, the life-time for each slice instance is configurable in our platform, e.g., according to the latency requirements of the transported traffic. Thus, applications with more stringent latency demands may have slices with longer life-times in order to reduce the frequency of performing the complete configuration-process.

To ensure low latencies, some applications need strict priorities over others. In this line, an experiment was conducted to demonstrate the flow isolation and resource assurance of our solution. Three slices were defined with strict serving priorities: low (5QI = 6), medium (5QI = 2), and high (5QI = 84) (see Table 5.7.4-1 in [20]). With this configuration, the slices with greater priority are always served before the ones with lower priority. We have injected UDP (User Data Protocol) flows in the medium priority slice at different bit-rates, namely, 100 Mbps, 2 Gbps, and 4 Gbps, by using the *iPerf* tool. In our IoV scenario, this bandwidth can be obtained considering the traffic aggregated from a set of vehicles connected to the slicing platform. Note that the maximum system transport capacity is ≈4 Gbps.

**Figure 9:**
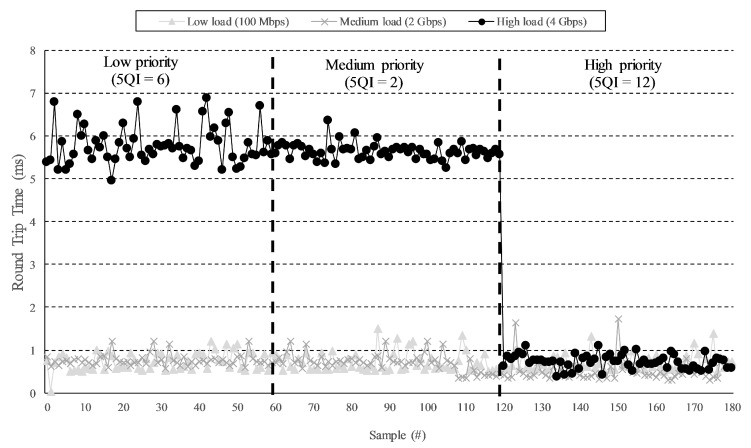
Round Trip Time (RTT) for critical traffic.

We measured the Round Trip Time (RTT) for critical low-bandwidth traffic of one message per second from the gateway to the app-server by transporting this traffic alternatively through the different instantiated slices. The experiment was configured as follows: during the first minute, the critical traffic was transported through the lowest priority slice; during the second minute, it was sent through the medium priority slice; and, finally, during the last minute, the critical traffic was tunneled through the highest priority slice. As shown in Figure 9, with a low background load in the system (100 Mbps), the RTT of the critical traffic was lower than 1 ms and independent of the slice assigned. A similar behavior was observed with a medium background load (2 Gbps) but, in this case, some measured values were far from the average, indicating a higher instability of the system. The last case, in which the system was supporting a high background load (4 Gbps), provided further insights. When the critical traffic was sent through the low or medium priorities slices, the RTT increased notably. In the former case, it was also noticeable that the jitter increased. Nevertheless, when the critical traffic was tunneled through the high priority slice, the RTT decreased to figures similar to those measured when the system was not saturated. Therefore, it can be seen how the application of strict priorities enables the slicing system to guarantee certain QoS metrics to critical services, even under high traffic load conditions. The attained RTT values are in-line with some of the state-of-the-art slicing proposals (e.g., [19]), or even improving other solutions (e.g., [25]).

**Figure 10:**
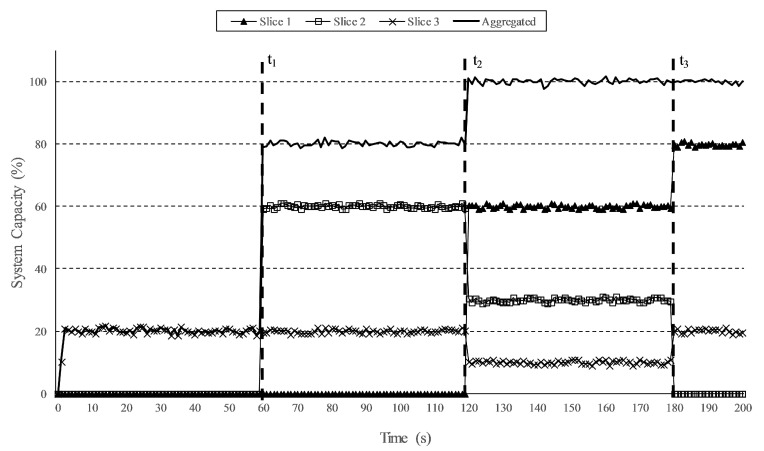
System capacity shared among slices.

Resource sharing is an interesting feature for services with non-strict QoS requirements, as it permits a higher utilization of the available network resources. In the following experiment, we defined three slices sharing the available system capacity (bandwidth). The capacity assigned to each slice, guaranteed and ceil (maximum ceiling), as percentage of the total system capacity, are shown in Table 1. Figure 10 shows the capacity utilization during the experiment in which the slices were dynamically receiving traffic. The experiment began with only Slice 3 transporting traffic, hence it took its maximum assigned system resources of 20%. At t = 60 s, Slice 2 received traffic. As the aggregated system capacity did not reach its maximum, both slices could use their respective ceil assigned resources, namely, 60% for Slice 2 and 20 % for Slice 3. At t = 120 s, Slice 1 started to transport traffic, thus it took its guaranteed resources of 60%. This provoked that both Slices 2 and 3 had to reduce their taken system capacities, thus they were reduced to their guaranteed ratios: 30% for Slice 2 and 10% for Slice 3. At this point, the aggregated system occupancy was 100%. Finally, at t = 180 s, Slice 2 stopped receiving traffic, thus its system utilization was reduced to 0%. This released resource could be assigned to the other slices, which reached their ceil bandwidths, i.e., 80% for Slice 1 and 20% for Slice 2, and the aggregated system utilization remained at 100%. With this strategy for the assignment of network resources, the slicing framework enabled a better utilization of the available system capacity. A similar experiment demonstrating resource sharing among slices was conducted in [26]. However, the considered conditions were much simpler, as the authors only studied the case of having two slices with neither minimum nor ceil throughputs set for each slice.

Finally, regarding the system-scalability, a stress test aiming at studying the system’s capability of managing a great number of simultaneously connected vehicles sending traffic was conducted. We emulated a series of LoRaWAN gateways connected to a common MEC-node, which provides access to the slicing system. To this end, we used the Lorhammer tool, which allowed us to emulate a given number of gateways with a series of attached end-devices generating traffic at a certain data-rate. We instantiated 1000 vehicular end-devices connected to each gateway and emulated up to 1000 gateways. This emulated a scenario serving vehicles in a whole geographical region. A similar scalability test was conducted in [27], but the authors focused on non-vehicular IoT scenarios and evaluated the attained signaling overhead instead of data-plane KPIs.

Considering the restrictions posed by the duty-cycle regulation in Europe (see Table I in [28]), each end-device generated a maximum of 84 packets of 50 bytes per hour. All traffic was tunneled by a common slice without bandwidth restrictions. Thereby, Table 2 shows the average throughput (packets/s) measured in the UPF as well as the average RTT between the emulated gateway and the app-server during a set of experiments with a duration of 1 h. Observe how the system could support the expected traffic load, even with a vast number of devices simultaneously connected and generating traffic at their maximum permitted rate. This peak load was achieved by emulating the attachment of 1000 LoRa GWs to the slicing system, which translates to 1 million devices connected to the system. The RTT results, under 1 ms, corroborate the low delay introduced by the system, even under a very heavy load.

## 6. Conclusions

5G will enable the development of novel IoV applications, including services in the areas of safety, traffic regulation and infotainment. However, traffic differentiation techniques are needed for providing these services with the highest level of quality to end-users. In this paper, we present a 5G network slicing framework that was validated and evaluated with different types of traffic and RATs, including current LTE and LoRaWAN technologies. It is based on a distributed MEC and cloud computing architecture, which enables the dynamic deployment in the cloud of VNFs and other micro-services along the end-to-end path from the end-device to the application server.

We show that our solution provides flow isolation and resource assurance for stringent applications as well as bandwidth distribution for more flexible services. The slicing framework has a reduced slice set-up time of less than 2.5 ms when the slice instance is already configured and around 240 ms when a new slice instance is created. The scalability of the network core was evaluated by emulating the connection of up to one million devices. These results clearly demonstrate the support of differentiated QoS requirements for vehicular-application data flows as diverse as safety services or autonomous maneuver commands (URLLC traffic), see-through services using augmented reality and high-definition video (eMBB), or mechanical monitoring (mMTC). These services can be offered simultaneously and their network resource allocation is ensured by a flexible priority-scheme.

Future lines of work include the development of a fine-grained slicing mechanism in the RAN, as a continuation to the slicing efforts applied in the edge and core networks, and using 5G new-radio (NR) communications. Moreover, we will also investigate the challenges of network slicing under roaming conditions, which is essential in vehicular scenarios. We expect to complement our advances in computing offloading [29] with network slicing capabilities in a whole 5G-enabled IoV ecosystem.

## Figures and Tables

**Table 1:** System capacity assigned to the slices.

No.	Guaranteed	Ceil
**Slice 1**	60%	80%
**Slice 2**	30%	60%
**Slice 3**	10%	20%

**Table 2:** Throughput and Round Trip Time (RTT) in the stress test.

Connections	Throughput (Packets/s)	RTT (ms)
1 GW (1000 devices)	10.75	0.709
10 GW (10,000 devices)	108.54	0.73
100 GW (100,000 devices)	1053.3	0.72
1000 GW (1,000,000 devices)	10,866.2	0.71

## Abbreviations

The following abbreviations are used in this manuscript:

4G	Fourth Generation (mobile/cellular networks)
5G	Fifth Generation (mobile/cellular networks)
CN	Core Network
DiffServ	Differentiated Services
DO	Domain Orchestrator
DSC	Dedicated Short-Range Communication
DSCP	Differentiated Services Code Point
eMBB	evolved Mobile Broadband
GO	General Orchestrator
IoT	Internet of Things
IoV	Internet of Vehicles
ITS	Intelligent Transportation Systems
LP-WAN	Low Power—Wide Area Network
MANO	Management and Orchestration
MEC	Multi-Access Edge Computing
mMTC	massive Machine-Type Communication
NFV	Network Function Virtualization
OBU	On-Board Unit
OvS	Open vSwitch
PHB	Per Hop Behavior
QFI	QoS Flow ID
QoS	Quality of Service
RAN	Radio Access Network
RAT	Radio Access Technology
RTT	Round Trip Time
SDF	Service Data Flow
SDN	Software Defined Network
SD-WAN	Software Defined—Wide Area Network
SCr	Slice Creator
SINR	Signal to Interference & Noise Ratio
SM	Slicing Manager
SSM	Slice Session Manager
TC	Traffic shaper and Controller
UDP	User Datagram Protocol
UDR	Unified Data Repository
URLLC	Ultra-Reliable, Low-Latency Communication
VIM	Virtualized Infrastructure Manager
VNF	Virtualized Network Function
